# Influence of anti-Nogo-A antibody treatment on the reorganization of callosal connectivity of the premotor cortical areas following unilateral lesion of primary motor cortex (M1) in adult macaque monkeys

**DOI:** 10.1007/s00221-012-3262-x

**Published:** 2012-09-19

**Authors:** Adjia Hamadjida, Alexander F. Wyss, Anis Mir, Martin E. Schwab, Abderaouf Belhaj-Saif, Eric M. Rouiller

**Affiliations:** 1 Program in Neurosciences, Department of Medicine, Faculty of Sciences and Fribourg Centre for Cognition, University of Fribourg, Chemin du Musée 5, 1700 Fribourg, Switzerland; 2 Novartis Pharma, Basel, Switzerland; 3 Brain Research Institute, University of Zürich and ETH Zürich, Zürich, Switzerland

**Keywords:** Reorganization, Callosal connectivity, Lesion, Tract-tracing

## Abstract

Following unilateral lesion of the primary motor cortex, the reorganization of callosal projections from the intact hemisphere to the ipsilesional premotor cortex (PM) was investigated in 7 adult macaque monkeys, in absence of treatment (control; *n* = 4) or treated with function blocking antibodies against the neurite growth inhibitory protein Nogo-A (*n* = 3). After functional recovery, though incomplete, the tracer biotinylated dextran amine (BDA) was injected in the ipsilesional PM. Retrogradely labelled neurons were plotted in the intact hemisphere and their number was normalized with respect to the volume of the core of BDA injection sites. (1) The callosal projections to PM in the controls originate mainly from homotypic PM areas and, but to a somewhat lesser extent, from the mesial cortex (cingulate and supplementary motor areas). (2) In the lesioned anti-Nogo-A antibody-treated monkeys, the normalized number of callosal retrogradely labelled neurons was up to several folds higher than in controls, especially in the homotypic PM areas. (3) Except one control with a small lesion and a limited, transient deficit, the anti-Nogo-A antibody-treated monkeys recovered to nearly baseline levels of performance (73–90 %), in contrast to persistent deficits in the control monkeys. These results are consistent with a sprouting and/or sparing of callosal axons promoted by the anti-Nogo-A antibody treatment after lesion of the primary motor cortex, as compared to untreated monkeys.

## Introduction

The motor cortical areas of macaque monkeys comprise four main distinct regions: the primary motor cortex (M1), the premotor cortex (PM), the supplementary motor area (SMA) and the cingulate motor area (CMA). These four cortical regions have been subdivided further into subareas on the basis of anatomical and functional criteria. For instance, the premotor cortex comprises a dorsal (PMd) area separated from the ventral (PMv) area (e.g. Kurata 1991, 1994a, b; Kurata and Hoffman 1994). The SMA has been subdivided into a rostral zone (pre-SMA) and a caudal zone (SMA-proper), based also on anatomical and functional criteria (e.g. Matsuzaka et al. 1992; Matsuzaka and Tanji 1996; Preuss et al. 1996; Inase et al. 1999; Shima and Tanji 1998, 2000; Liu et al. 2002; Fuji et al. 2002; Russo et al. 2002; Akkal et al. 2007; Nakajima et al. 2009). Based essentially on the topographic distribution of corticospinal neurons, three distinct subareas have been recognized in the CMA (Dum and Strick 1991; He et al. 1995). Finally, M1 exhibits different properties in its caudal versus rostral portion (e.g. Strick and Preston 1982; Friel et al. 2007; Rathelot and Strick 2009). In intact monkeys, the corticocortical connectivity of the motor cortical areas is well established, both within the same hemisphere (e.g. Luppino et al. 1990, 1993, 1999, 2003; Kurata 1991; Matelli et al. 1998; Tanné-Gariépy et al. 2002a) and with the other hemisphere (e.g. Jenny 1979; Rouiller et al. 1994a; Liu et al. 2002; Marconi et al. 2003; Boussaoud et al. 2005). The multiplicity of motor cortical areas in primate represents a basis for a (partial) vicarious substitution of one area by others in case of a restricted lesion affecting a specific motor zone. Along this line, it was shown that PM plays a crucial role in the spontaneous (incomplete) functional recovery of hand dexterity after M1 lesion (e.g. Liu and Rouiller 1999; Frost et al. 2003; Dancause et al. 2006), for instance based on an enhancement of the homolateral connectivity of PM with the postcentral gyrus as a result of M1 lesion (Dancause et al. 2005).

The study of Dancause and collaborators (2005) demonstrates that the intra-hemispheric connectivity of a motor cortical area (PM in the present case) is subjected to spontaneous and significant changes, such as rewiring, as a result of M1 lesion. Such rearrangement is most likely, at least in part, the anatomical support for (incomplete) spontaneous recovery from the lesion. If this is the case, one may predict that a treatment enhancing functional recovery after motor cortical lesion is likely to promote adaptive changes in corticocortical connectivity. In this context, the present study aims at testing the hypothesis that, after a lesion of M1, the callosal connectivity of PM is modified, possibly preserved and/or enhanced as a result of a treatment promoting better functional recovery, by neutralizing the neurite growth inhibitor Nogo-A. Such intervention has been shown to enhance functional recovery and sprouting of the corticospinal tract in rats (see Gonzenbach and Schwab 2008; Schwab 2010 for review) and in monkeys subjected to spinal cord injury (Fouad et al. 2004; Freund et al. 2006, 2007, 2009). More specifically, we hypothesize that after M1 lesion, in the absence of treatment, there is a limited spontaneous sprouting of the callosal projections reaching the ipsilesional PM. In contrast, the administration of an anti-Nogo-A antibody may preserve connectivity and/or promote local sprouting of callosal fibres reaching PM rostral to the lesioned M1. To test this hypothesis, adult macaque monkeys were subjected to a unilateral lesion of M1, subdivided then in a group of monkeys without treatment and a group of monkeys treated with an anti-Nogo-A antibody. In both groups of monkeys, after (incomplete) functional recovery, the anatomical tracer BDA was injected in the ipsilesional PM to quantify the number of callosal retrogradely labelled neurons in the opposite (intact) hemisphere projecting to PM. Although the BDA injection sites were located mainly in PMd, some sites also involved PMv. For this reason, we will use below the common nomenclature PM to designate these two premotor areas. We hypothesize that, after M1 lesion and due to preservation of connectivity and/or increase in axonal sprouting, more labelled callosal neurons will be found in the anti-Nogo-A antibody-treated monkeys than in the untreated monkeys, after injection of BDA in the ipsilesional PM.

## Materials and methods

The inter-hemispheric connectivity of PM has been derived from 8 adult macaque monkeys (*Macaca fascicularis;* see Table 1). The experiments were conducted on two groups of monkeys: (1) four control monkeys (lesion of M1, no treatment) and (2) four treated monkeys (lesion of M1 and anti-Nogo-A antibody treatment). In one treated monkey (Mk-LA), the delivery of anti-Nogo-A antibody had to be interrupted after 2 weeks instead of the standard treatment duration of 4 weeks, due to an infection generated by the subcutaneous catheter. This animal (Mk-LA) was thus excluded from further analysis, reducing the group of anti-Nogo-A antibody-treated monkeys to 3 individuals. All monkeys were subjected to injections of the neuroanatomical tracer BDA (biotinylated dextran amine) in PM. In the present report, the analysis was restricted to the neurons retrogradely labelled with BDA in the hemisphere opposite to the injected PM. BDA is generally used for anterograde tracing with the aim, in the present series of experiments, to study the efferent projections originating from PM (data will be reported elsewhere). Nevertheless, BDA was found to provide retrograde labelling as well, yielding consistent and reliable data, as seen in previous studies in which several retrograde tracers, including BDA, were switched around across cases (Rouiller et al. 1999; Tanné-Gariépy et al. 2002a, b; Liu et al. 2002; Morel et al. 2005; Boussaoud et al. 2005). Based on these previous data supporting the reliability of BDA for retrograde tracing, the present analysis was conducted to assess the origin of callosal projections reaching PM. Table 1 shows a survey of the parameters of BDA injections in the premotor cortex and ibotenic acid injections to induce a permanent lesion in M1. Surgical procedures and animal care were conducted in accordance with the Guide for the Care and Use of Laboratory Animals (ISBN 0-309-05377-3; 1996). The experimental protocol was approved first by the local (cantonal) ethical committee (surveying animal experimentation). Finally, the experiments were authorized by the cantonal (Fribourg) and federal (Swiss) veterinary officers. The present experiments were covered by the following authorizations: FR 24/95/1; FR 44/92/3; FR 157/01, FR 157/03, FR 157/04, FR 156/04, FR 156/06, FR 157e/06; FR 185-08.

**Table 1:** Summary of the properties of each monkey included in the study

Monkey groups	Untreated				Treated with anti-Nogo-A antibody			
	Mk-GE	Mk-RO	Mk-CE	Mk-BI	Mk-VA	Mk-SL	Mk-MO	Mk-LA^b^
Species	fasc	fasc	fasc	fasc	fasc	fasc	fasc	fasc
Lesion of M1 hand area	Yes	Yes	Yes	Yes	Yes	Yes	Yes	Yes
Treatment	None	None	None	None	Anti-Nogo-A antibody	Anti-Nogo-A antibody	Anti-Nogo-A antibody	Anti-Nogo-A antibody
Age at time of lesion (rounded 0.5 year)	5	4	4.5	5	5.5	5.5	5.5	5
Weight at time of lesion (kg)	2.8	3.2	3.8	5	4.9	4.6	5.6	2.6
Volume of ibotenic acid injected (μL)	13	18	40	29.74	15.5	18	20	13.5
No. of ICMS sites injected with ibotenic acid	13	12	21	29	11	11	20	9
Volume of BDA injected (μL)	2.1	4.8	16	7.2	5	10	10.8	2
No. of BDA sites injected	5	6	16	11	5	10	12	5
Hemisphere in which BDA was injected^a^	LH	LH	LH	LH	LH	LH	LH	LH
Time interval between lesion and beginning of post-lesion ICMS (months)	3.25	5	11	7.3	10.5	16.5	3.2	6.75
Time interval between end of post-lesion ICMS and BDA injection (days)	17	30	12	27	14	58	75	160
Duration of survival time (days)	29	21	21	22	22	18	21	27
Cortical areas of BDA injections	PMd/PMv	PMd	PMd/PMv	PMd/PMv	PMd/PMv	PMd/PMv	PMd/PMv	PMd/PMv
Volume of injection site core (in mm^3^)	23.8	41.4	64.2	83.6	21.1	4.2	106.3	17.8
Total number of callosal-labelled cells	167	89	76	958	927	49	1,805	73
Total volume of lesion (mm³) Grey matter (motor cortex + post-central gyrus)	48.7	14	112.8	20.13	20	78.2	41.8	3.12
Volume of lesion in post-central gyrus (mm³)	7.6	0	10.1	0	5.8	1.8	0	0
Volume of lesion spread to subcortical white matter (mm³)	0	0	86.5	0	0	130.6	0	0

Under species, “fasc” is for *Macaca fascicularis*

Monkey Mk-CE was part of a pilot study (Liu and Rouiller 1999), with the initial aim to generate a large lesion. In subsequent monkeys (Mk-GE, Mk-LA and MK-VA), the volume of ibotenic acid was reduced to generate a lesion more focused to the M1 hand area. In the monkeys included later over the 8 years of the study (Mk-RO, Mk-BI, Mk-MO, and Mk-SL), ibotenic acid was injected under propofol anaesthesia (as required by new ethical guidelines), instead of in the awake state in the preceding monkeys. The volume of ibotenic acid was thus slightly augmented, as propofol may reduce the excitotoxic effect of ibotenic acid (Snyder et al. 2007)

^a^Ipsilesional hemisphere (LH = left hemisphere)

^b^Mk-LA was excluded from further analysis, as the anti-Nogo-A antibody treatment lasted only 2 weeks (instead of 4 in all other treated animals) and because the lesion turned out to be very small

In the animal facility, monkeys were housed in rooms of 12 m^3^, in which usually 2–4 monkeys were free to move and to interact among each other.^1^ Before daily behavioural testing in the morning, the animal caretaker transferred the monkeys to temporary cages, for subsequent transfer to a primate chair, in which the monkeys were transported to the behavioural laboratory. The monkeys had free access to water and were not food deprived. The reward (pellets) obtained during the behavioural tests was the first daily access to food. After completion of the behavioural tests, the monkeys received additional food (fruits and cereals). The body weight of the animals was monitored on each working day. In case the body weight dropped by 10 % or more, the experiment was interrupted until the monkey regained the lost weight (this criterion for interruption was not met in the course of the present experiments).

The survey of the temporal sequence of the overall experimental protocol conducted on each monkey is as follows. (a) The monkeys were subjected to initial behavioural training to several manual dexterity tasks during several months (Schmidlin et al. 2011), until reaching a pre-lesion plateau of performance. The duration of the pre-lesion behavioural training was quite variable across monkeys, reflecting different inter-individual capabilities to consolidate a wide palette of manual dexterity tasks. Furthermore, for scheduling reasons, the (stable) pre-lesion plateau phase was prolonged in some cases to wait for an adequate time widow to conduct the subsequent, intensive daily pre-lesion ICMS sessions (see next step). (b) The hand representation in M1 was identified electrophysiologically based on intracortical microstimulation (ICMS). (c) A permanent lesion of the hand area in M1 was performed by infusing ibotenic acid, immediately followed in the group of treated monkeys by infusion of anti-Nogo-A antibody during 4 weeks. (d) The daily behavioural assessment of manual dexterity was pursued during several weeks or months (depending on the individual time course of recovery: see Table 1), in order to assess the functional deficit and the progressive (incomplete) restitution of manual performance, until a post-lesion plateau was reached. (e) The ICMS sessions were repeated at the same cortical sites as pre-lesion, in order to establish motor map changes observed post-lesion and possibly related to the extent of functional recovery (ICMS data reported elsewhere). (f) To investigate the possible roles played by different motor cortical areas (e.g. PMd or PMv) in the functional recovery post-lesion, reversible inactivation sessions were conducted by infusing muscimol (GABA agonist) at sites previously established using ICMS (muscimol data reported elsewhere). (g) The tracer BDA was finally injected in PM, to establish its post-lesional connectivity, which is the topic of the present study regarding the origin of callosal afferents to the ipsilesional PM. (h) After a survival time (see Table 1) to allow axonal transport of BDA, the monkeys were killed for histological evaluation.

The present experimental protocol, described below step by step in more detail, applies to the group of treated monkeys (Mk-VA, Mk-SL, Mk-MO, Mk-LA), whereas for the group of control monkeys (Mk-GE, Mk-RO, Mk-CE and Mk-BI), the anti-Nogo-A antibody treatment was omitted. The 7 cases considered for analysis (Mk-GE, Mk-RO, Mk-CE, Mk-BI, Mk-VA, Mk-MO and Mk-SL) have already been considered in recent reports dealing with the influence of the lesion of M1 on the manual performance of the ipsilesional hand or in relation to adult progenitor cells transplantation (Kaeser et al. 2010, 2011; Bashir et al. 2012).

### Surgery

The monkeys were implanted unilaterally with a chronic, stainless steel or tecapeek chamber giving access to M1, the dura mater being, however, left in place (see Schmidlin et al. 2004 for detail). The monkeys were sedated with i.m. injection of ketamine (Ketalar, 5 mg/kg) and pre-medicated as previously described, in particular with injection of the analgesic carprofen (Rymadil, 4 mg/kg, s.c.) to reduce pain after surgery (Schmidlin et al. 2005; Wannier et al. 2005; Freund et al. 2006). The surgical intervention itself was conducted under aseptic conditions and deep anaesthesia, maintained several hours by i.v. infusion of propofol (mixture of 1 % propofol and 4 % glucose in saline, 1 volume of propofol and 2 volumes of glucose delivered at the rate of 0.1 ml/min/kg). Ketamine was added to the perfusion solution, as previously reported (Freund et al. 2007). The surgery was carried out under continuous monitoring of the following parameters: heart rate, respiration rate, expired CO_2_, arterial O_2_ saturation and body temperature. After surgery, the animals were treated with antibiotics (ampicilin 10 %, 30 mg/kg, s.c.) and analgesics (pills of Rymadil mixed with food) during several days. The chambers were fixed to the skull with titanium screws and orthopaedic cement (Palacos). The inside of the chronic chamber was cleaned daily with Betadine, and an antibiotic ointment was spread on the dura mater surface to reduce the risk of infection.

### Behavioural assessment: Brinkman box

**Fig. 1:**
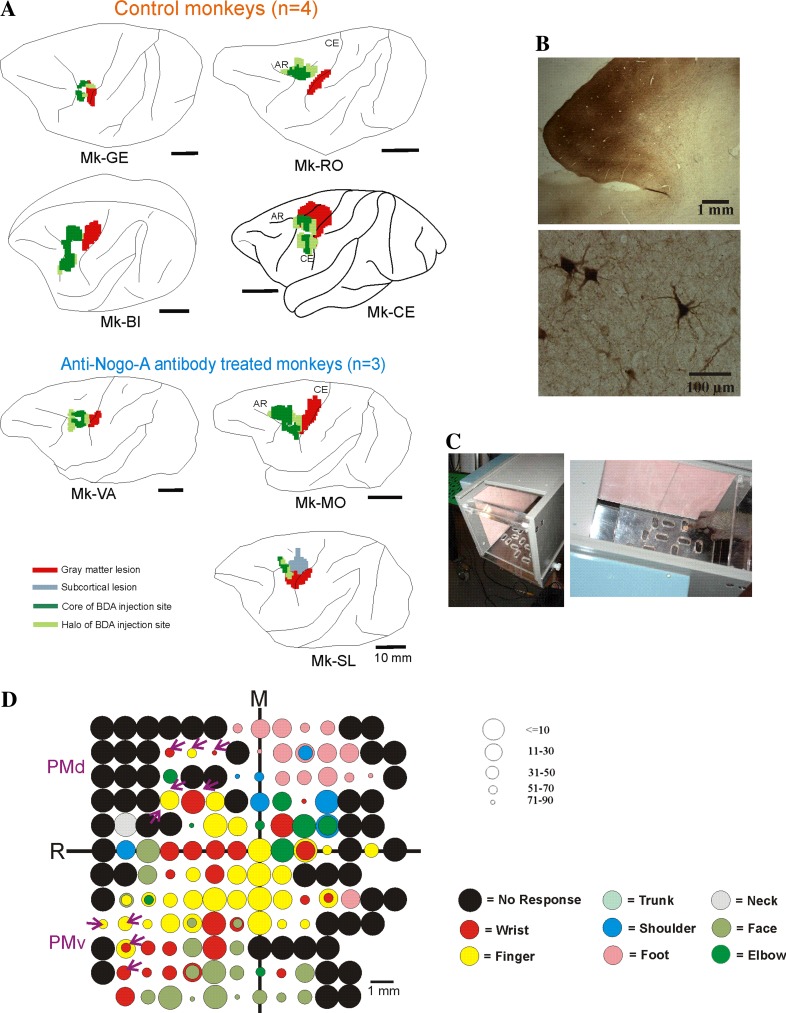
**a** Reconstruction on a lateral view of the *left* hemisphere in each monkey of the location and extent of the lesion aimed at the M1 hand representation (*red* territory) and BDA injection sites (territory in *dark green* for the core of the injection site and *light green* for the halo), as seen in transparency of the cortical surface. The actual volume of the lesions and BDA injections sites are given in Fig. 6b and Table 1. Mk-LA is not shown as it was excluded from the neuroanatomical analysis (see Table 1). **b** Photomicrograph of a typical BDA injection site in PMd and resulting BDA retrogradely labelled callosal cells in the opposite hemisphere. **c** View of the “Brinkman box” used for behavioural assessment of manual dexterity, representing a variation in our modified Brinkman board task (see text). The *left* picture shows the open facet of the “box” allowing access to the 20 wells, in which the pellets are placed. The *right* picture shows that “box” from above with the monkey’s hand aimed to a well filled with a pellet to be grasped using the precision grip between the index finger and the thumb. At the beginning of the test, the 20 wells are each filled with a pellet. As the top facet of the “box” is transparent, the hand movements are executed under visual control. The behavioural test was taped with a video camera placed below the “box”. **d** Raw ICMS map established post-lesion in Mk-BI, as it represented the basis to identify positions where to perform penetrations with the syringe to infuse muscimol in PMd or PMv and then injections of BDA. Along the axes, “R” is for rostral and “M” is for medial. The *circles* represent the positions of electrodes penetrations for ICMS on the cortical surface, with *colour code* to represent the body territory activated at the lowest threshold along the corresponding electrode penetration. The threshold value is given by the size of the *circle* in microAmps (see on the *right* of the ICMS map). *Circles* comprising 2 colours are for ICMS electrode penetrations along which 2 body territories were activated at comparable threshold values. The *purple arrows* are for the sites of infusion of muscimol in PMd or in PMv, which were then used also as sites for BDA injections. Six sites of BDA injections were selected in PMd and 5 sites in PMv. BDA was injected in PMd and in PMv at sites where digits were activated by ICMS (*yellow circles*), or at sites where ICMS elicited at lowest threshold a wrist movement (*red circles*). In the latter case (wrist representation), there was also some ICMS effect on the digits, but at higher threshold. Note in M1 the presence of a few ICMS sites where digits movements were elicited at low intensity, but much fewer than on the pre-lesion ICMS map (not shown). Note also the presence of numerous non-micro-excitable sites, typical of the post-lesion ICMS map

**Fig. 2:**
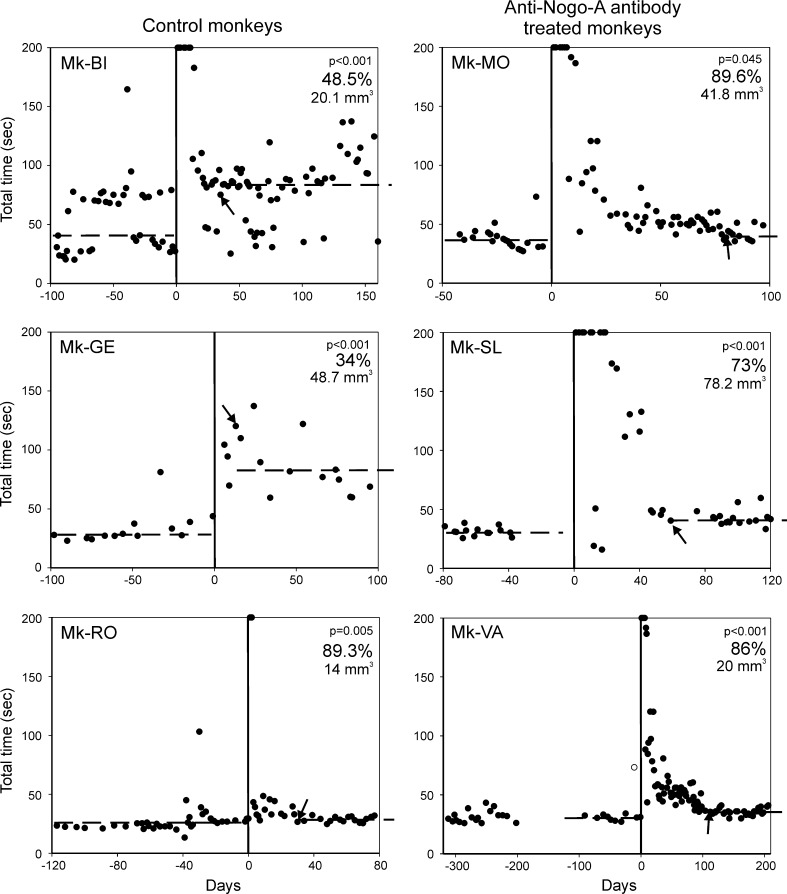
Behavioural data showing the manual dexterity of six monkeys before the lesion (negative days on the abscissa) and post-lesion (positive days on the abscissa). The day of the lesion (day zero) is indicated by a *vertical line*. Each *dot* value corresponds to the total time (in seconds) needed by the monkey in a given session to empty the board containing 20 wells using the contralesional hand (see Fig. 1c). The median total time pre-lesion is given by the *horizontal dashed line* on the *left* part of each graph. Note the dramatic increase in total time immediately after the lesion, reflecting the deficit (increased difficulty to grasp the pellet using the precision grip). The values saturated at 200 correspond to sessions immediately post-lesion in which the monkey was unable to complete the task (empty the 20 wells). Then, there is a progressive recovery of manual dexterity. The recovery was considered as complete on the day indicated by the *arrow*, corresponding to the time point at which the monkey reached a plateau in the modified Brinkman board task (see Kaeser et al. 2010, 2011). The median total time at plateau of recovery is indicated by the *horizontal dashed line* on the *right*. In all monkeys, the post-lesion median total time was significantly longer than the pre-lesion median total time (*p* value given for each graph). The percentage of functional recovery was calculated by dividing the pre-lesion median total time by the post-lesion median total time at plateau, multiplied by 100. The percentage of functional recovery is given in each graph. For the interpretation of the data (see text), the volume of the cortical lesion in mm^3^ is indicated also in the graphs for each monkey. Note that Mk-BI exhibited a peculiar, bimodal behavioural pattern for this manual dexterity task, present, however, both pre-lesion and post-lesion: in some daily sessions, the monkey completed the task quickly (in about 25 s pre-lesion), whereas in other daily sessions, it took clearly more time (about 70–80 s pre-lesion as well). As the same bimodal distribution of total times was maintained post-lesion, it allows comparison of the median values. In line with a higher median value observed post-lesion for Mk-BI, note that for each of the two behavioural patterns, the variability across daily sessions was larger post-lesion as compared to pre-lesion, suggesting that the monkey was less regular and less comfortable with the task after the lesion. Moreover, pre-lesion Mk-BI exhibited the “quick” and the “slow” behaviours in equal proportions (50/50 %). After lesion (at plateau), the “slow” behaviour was more frequently present (73 % of the daily sessions) than the “quick” behaviour (27 %), also supporting the strong deficit induced by the lesion

The monkeys were trained to perform a large palette of manual dexterity tasks, as previously described in detail (e.g. Rouiller et al. 1998; Freund et al. 2009; Kaeser et al. 2010, 2011; Schmidlin et al. 2011; see also http://www.unifr.ch/neuro/rouiller/motorcontcadre.htm). Our main behavioural test was based on a modified version of the Brinkman board task (e.g. Brinkman and Kuypers 1973; Brinkman 1984), in which the monkeys had to grasp food pellets from wells using the precision grip (opposition of thumb and index finger). The behavioural data for all monkeys subjected to motor cortex lesion will be presented in a comprehensive manner elsewhere (based on the complete palette of tests and on a larger populations of monkeys, including those not subjected to BDA injections in the premotor cortex). The present report will show behavioural data only for monkeys subjected to a lesion of the motor cortex and to BDA injection in the premotor cortex. In the present report, the behavioural assessment is focused on a further variation in the modified Brinkman board task (Schmidlin et al. 2011), consisting of a “box” containing only 20 wells (instead of 50) but confined to a restricted volume, limiting the movements and orientations of the hand to approach the wells (Fig. 1c). The dimensions of the “box” are 23 cm (length) × 13 cm (width) × 10 cm (height). Only one side of the “box” is open to allow access with the hand to the pellets inside the “box”. The board inside the “box” is made of 10 wells oriented vertically and 10 wells oriented horizontally. Under visual control, the monkey had to grasp the food pellets. The total time (in seconds) needed to empty all 20 wells was measured (Fig. 2). After several weeks of training, the monkeys reached a plateau of hand performance, after which a pre-lesion performance of reference was established (Fig. 2), before the motor cortex was electrophysiologically mapped and lesioned (see below). The behavioural assessment was continued during the weeks/months post-lesion, in order first to quantify the deficit of manual dexterity induced by the lesion of M1 and then to establish the time course of (incomplete) functional recovery, until a post-lesion behavioural plateau was reached. As previously reported (e.g. Kaeser et al. 2010, 2011; Bashir et al. 2012; Schmidlin et al. 2011), the time course of functional recovery varied across monkeys, explaining variable time intervals between the cortical lesion and the onset of the post-lesion ICMS experiments (see Table 1), in addition to schedule (seasonal) constraints. Mk-CE is not included in the behavioural data based on the Brinkman box task (Fig. 2) because, when the experiment was conducted on Mk-CE, the Brinkman box test was not introduced yet in our behavioural palette of manual dexterity tasks. However, a comparison of the functional recovery observed in the six monkeys engaged in the Brinkman box task (Fig. 2) with that derived in Mk-CE from the modified Brinkman board task is shown (see Fig. 6c and related text in the results section).

### Electrophysiology: intracortical microstimulation (ICMS)

Electrophysiological intracortical microstimulation (ICMS) sessions were performed twice, first before lesion in order to guide the lesion procedure by mapping the M1 hand representation and, second, several months after the lesion when the monkeys reached a stable manual performance in the motor tasks (see e.g. Rouiller et al. 1998; Liu and Rouiller 1999; Schmidlin et al. 2004; Freund et al. 2006, 2009). A tungsten microelectrode (0.1–1 MΩ impedance, FHC Inc, USA.) was used to micro-stimulate the motor cortex accessible from the chronic chamber, along penetrations performed at 1 mm distance from each others (see e.g. Schmidlin et al. 2004, 2005). Along each electrode track, ICMS was applied below the surface of the dura at intervals of 1 mm, along a variable distance depending on the position of the penetration with respect to the central sulcus (see Kaeser et al. 2010). The effect of ICMS was assessed by visual inspection and/or palpation of the body part (articulation) at which a movement was elicited. The minimal current (ICMS threshold) producing this movement was determined at each stimulation site. A raw ICMS map was reconstructed first, showing the position of the electrode penetrations on the surface of the cerebral cortex (as shown in Fig. 1d for Mk-BI). In a second step, an unfolded ICMS map was established, as previously reported (Kaeser et al. 2010: supplementary Fig. 1; Park et al. 2001, 2004), representing a further basis to guide injections of ibotenic acid to produce a permanent lesion of M1 targeted mainly on the hand area (pre-lesion ICMS sessions) and BDA injections in PM (post-lesion ICMS sessions). As expected, in the hand representation in M1, along most electrode penetrations performed pre-lesion, the lowest current (threshold) eliciting movement of the contralateral digits was below 10 μAmps. In contrast, in PM along individual electrode penetrations considered for BDA injections, the lowest thresholds to elicit contralateral digit movements ranged between 25 and 80 μAmps, with the exception of an ICMS site with a threshold at 17 μAmps (the detailed ICMS maps obtained post-lesion will be reported elsewhere).

### Permanent lesion of M1 hand representation with ibotenic acid

In order to induce a permanent lesion of the motor cortex, the neurotoxic ibotenic acid (10 μg/μl in phosphate buffer) was infused using a Hamilton micro-syringe at selected intracortical microstimulation (ICMS) sites of the hand area in M1 unilaterally, as previously reported in detail (Liu and Rouiller 1999; Kaeser et al. 2010, 2011; Bashir et al. 2012; Peuser et al. 2011). The number of ICMS sites injected and the total volume of ibotenic acid infused in M1 were determined based on the extent and shape of the hand area established for each monkey on the basis of the ICMS map. As the extent of the hand area varied across monkeys, the volume of ibotenic acid injection was adapted to this parameter (Table 1). In one monkey (Mk-CE), performed in the context of a pilot study long time ago (Liu and Rouiller 1999), a large volume of ibotenic acid was injected in M1, generating an extensive lesion. For subsequent animals, in order to produce a lesion more focused to the hand representation in M1, the volumes of ibotenic acid injected were reduced (Table 1), ranging from 13 to 30 μl depending on the extent of the hand area determined by ICMS. The parameters of ibotenic acid injections are indicated for each monkey in Table 1. As a result of ibotenic acid infusion, the contralateral hand exhibited after a few minutes a dramatic flaccid paralysis. The location and extent of the lesion are shown on lateral views of the left hemisphere in the four untreated (control) and the three anti-Nogo-A antibody-treated monkeys included in the neuroanatomical analysis (Fig. 1a).

### Anti-Nogo-A antibody treatment

As outlined in Table 1, four of the eight monkeys subjected to unilateral permanent lesion of the motor cortex were treated with anti-Nogo-A antibody, delivered immediately after the lesion during four weeks (except in Mk-LA in which the treatment lasted only 2 weeks and eliminated from further analysis). The anti-Nogo-A antibody treatment was similar to that applied to monkeys subjected to spinal cord injury (Freund et al. 2006, 2007, 2009), except that two osmotic pumps were implanted instead of one. The tube delivering the antibody from one osmotic pump was placed intrathecally in the cervical cord, whereas the tube of the second osmotic pump was placed below the dura in the vicinity of the lesion of M1. The anti-Nogo-A antibody treatment was tested here for motor cortex lesion in monkeys, as it was found to significantly enhance functional recovery after cortical lesion in rats (Papadopoulos et al. 2002; Emerick et al. 2003; Emerick and Kartje 2004; Markus et al. 2005; Seymour et al. 2005; Tsai et al. 2007; Cheatwood et al. 2008; Gillani et al. 2009). Delivery of the anti-Nogo-A antibody in the cervical cord, in addition to the cerebral cortex, was motivated by possible enhancement of sprouting of corticospinal axons originating from PM at segmental level, in line with evidence for an increase in functional recovery and sprouting of corticospinal axons after cervical cord injury in macaques treated with anti-Nogo-A antibody (Freund et al. 2006, 2007, 2009).

### BDA injection and histology

After completion of the post-lesion ICMS investigations (see Fig. 1d for Mk-BI), reversible inactivation experiments (muscimol infusions) were conducted to investigate the possible role played by distinct motor cortical areas in the functional recovery (data to be reported elsewhere). Depending on the duration of the reversible inactivation experiments in each monkey, corresponding to a variable time interval as indicated in Table 1, BDA was injected in the ipsilesional PM, following a surgical procedure and anaesthesia protocol as described above. BDA (MW 10′000, 10 % in phosphate-buffered saline, Molecular Probes, Eugene, OR) was injected unilaterally into PM (mainly in PMd and to a lesser extent in PMv) on the lesioned hemisphere of the 8 lesioned monkeys (except in Mk-RO in which the injection was restricted to PMd, as PMv was not accessible from the implanted chronic chamber), using a Hamilton micro-syringe, as previously described (e.g. Rouiller et al. 1994a, b, 1996, 1999, 2003; Liu et al. 2002; Tanné-Gariépy et al. 2002a; Boussaoud et al. 2005; Morel et al. 2005; Cappe et al. 2007, 2009). PMd and PMv areas on the ipsilesional side were identified partly based on the ICMS sessions performed pre-lesion and post-lesion. As illustrated for Mk-BI (Fig. 1d), the post-lesion ICMS map exhibited two territories, one located dorso-rostrally and one ventro-rostrally, most likely corresponding to PMd and PMv, respectively, as reflected by the higher thresholds observed than in M1 (see above for threshold ranges). Sites (or in the vicinity) where ICMS produced digits and/or hand (wrist) movements were then selected for BDA injections (purple arrows in Fig. 1d, pointing to 6 injection sites in PMd and 5 injection sites in PMv). When the ICMS assessments were less extensive in PM, the position of PMd and PMv was also estimated based on their standard (expected) position with respect to the well-delineated M1 hand area (as derived from numerous previous studies: e.g. Boussaoud et al. 2005; Morel et al. 2005). The injections of BDA were preceded by the reversible inactivation sessions, in which muscimol was infused in PMd and/or PMv. As a result of muscimol infusion, there was in most cases a drop of the manual performance recovered post-lesion, suggesting that the inactivated areas play a role in the motor control and in the functional recovery. This is consistent with the idea that the areas infused with muscimol correspond to PMd and/or PMv. In most cases, the sites infused with muscimol are the same loci where BDA was injected at a later time point.

The detailed parameters of BDA injections are given for each monkey in Table 1. The survival time after BDA injection ranged from 18 to 29 days, to allow anterograde transport of the tracer down to the cervical cord, as assessed in previous experiments (Rouiller et al. 1994a, b, 1996). Such duration of survival time is also sufficient for retrograde transport of BDA to the intact hemisphere. The animals were then killed with an overdose of pentobarbital sodium (90 mg/kg body weight, i.p.). Transcardiac perfusion with 0.9 % saline (500 ml) was followed by fixative (paraformaldehyde 4 % in phosphate buffer), and 10, 20, 30 % solutions of sucrose in phosphate buffer. The brains were placed in a 30 % solution of sucrose (in phosphate buffer) for cryoprotection during 3–5 days. Frontal sections (50 μm thick) of the brain were prepared using a cryostat and collected in five series. BDA staining was revealed in one series of sections, as previously described (Rouiller et al. 1994a, b). A second series of sections was Nissl stained with cresyl violet, whereas a third series of sections was processed to visualize the marker SMI-32, as previously described (Liu et al. 2002; Wannier et al. 2005; Beaud et al. 2008). The two remaining series of sections were kept in reserve.

### Data analysis

Nissl- and SMI-32-stained sections were used to reconstruct on consecutive sections the position and extent of the permanent lesion in M1. BDA-stained sections were used to reconstruct the position and extent of BDA injection sites in PM. The lesion and the BDA injection sites were positioned on a lateral view of the lesioned hemisphere (Fig. 1a). An individual BDA injection site appears as a column of about 1–3 mm of diameter. As BDA was injected at multiple sites adjacent to each other, a composite BDA injection site was obtained, corresponding to a dense and dark centre zone (“core” of the injection site), surrounded by a less dense zone (“halo” of the injection site; Fig. 1b). The core and “halo” of the composite BDA injection site are represented for each monkey by the dark green and light green areas in Fig. 1a, respectively. Applying a comparable approach as previously described in detail (see e.g. Pizzimenti et al. 2007; Darling et al. 2009), based on an ad hoc function of the Neurolucida software (based on the Cavalieri method; Microbrightfield, Colchester, VT), the volume of the cortical lesion (in mm^3^) was extrapolated from the reconstructions of the lesion on consecutive SMI-32-stained histological sections of the brain taken every 0.25 mm intervals (see Table 1). The same procedure was used to determine the volume of the BDA injections sites on BDA-stained sections, separately for the core and the halo territories. On the corresponding histological sections (SMI-32, BDA staining), the lesion territory, the core of the BDA injection site and the halo of the BDA injection site were each delineated by tracing closed contours on their estimated boundaries with different colours in Neurolucida. The consecutive sections were then aligned along the rostro-caudal axis, using the mark left by a needle inserted through the brain before sectioning. The Cavalieri estimator was then applied to estimate the total area and the volume of the territories identified as different objects. The measurement of volumes (lesion territory or BDA injection sites) were not corrected for potential tissue shrinkage, assuming it was most likely comparable across monkeys as the histological processing was similar from one animal to the next.

The distribution of retrogradely BDA-labelled neurons on the opposite hemisphere was plotted using Neurolucida software, on sections taken at 1 mm interval. Drawings with plots of labelled neurons were then printed in the form of pdf files for later processing using the software CorelDraw 14 (X4). As previously reported in tracing studies in which all labelled neurons were counted on the analysed sections (see e.g. Lavenex et al. 2000; Geuna 2000; Benes and Lange 2001), stereological technique is not adequate. Stereology is appropriate when the number of cells to be counted is so large that samples have to be considered. In the present study, all labelled neurons were systematically chartered (as the number of labelled neurons in the intact hemisphere was relatively low). This approach is considered as adequate because all samples were analysed using the same procedure.

To account for variability due to the size of BDA injection sites and BDA uptake, the number of BDA-labelled neurons was normalized based on the volume of the core of the corresponding BDA injection site: the number of BDA-labelled cells was divided by the volume of the core of the injection site expressed in mm^3^. Figure 1b illustrates a typical BDA injection site in PMd (top panel) and retrogradely BDA-labelled callosal neurons in the opposite hemisphere (bottom panel). The parcellation of the cerebral cortex in the intact hemisphere, in which the labelled neurons were distributed into distinct cortical areas, was based on criteria previously defined (Liu et al. 2002; Boussaoud et al. 2005).

## Results

The lesion of the motor cortex was targeted to the hand representation of M1 (as determined on the basis of ICMS data), which varied in size and precise position across monkeys. As a result, there was also inter-individual variability in terms of size and location of the lesion, as represented in transparency of the brain surface in the left hemisphere of the seven monkeys subjected to a lesion and included in the analysis (the red areas in Fig. 1a correspond to the extent of grey matter affected by the lesion).

### Preliminary behavioural data

The behavioural consequence of the lesion affecting the hand area in M1 was a dramatic paralysis of the opposite hand, within the minutes following the infusion of ibotenic acid (Liu and Rouiller 1999). In the present study, the behavioural assessment based on the Brinkman box task was conducted on six monkeys subjected to a substantial lesion of M1, followed by a significant deficit of manual dexterity (Fig. 2). In the group of control monkeys (Mk-BI, Mk-GE and Mk-RO), two of them showed a considerable increase in the total time needed to empty the 20 wells containing the pellets after the lesion, a deficit that was long lasting: the post-lesion recovery was clearly incomplete, representing 48.5 % of the original (pre-lesion) performance in Mk-BI and 34 % in Mk-GE (Fig. 2 left column). The control monkey Mk-RO exhibited a very good spontaneous recovery post-lesion, but the volume of the lesion was clearly smaller than in the other two control monkeys. In the subgroup of the anti-Nogo-A antibody-treated monkeys, there was a post-lesion recovery ranging from 73 to 89.6 % (Fig. 2 right column), although the volume of the cortical lesion affecting the grey matter was comparable (Mk-VA; Mk-MO) or even larger (Mk-SL) than in the two control monkeys (Mk-BI; Mk-GE) exhibiting clearly incomplete functional recovery (below 50 %). Taking into account that the good spontaneous recovery observed in Mk-RO is most likely related to a restricted lesion, the behavioural data for the other five lesioned monkeys (Fig. 2) provide preliminary evidence for a better functional recovery in the subgroup of anti-Nogo-A antibody-treated monkeys (to be confirmed on a larger number of monkeys and additional behavioural tests).

### Callosal connectivity of PM in the untreated (control) monkeys

**Fig. 3:**
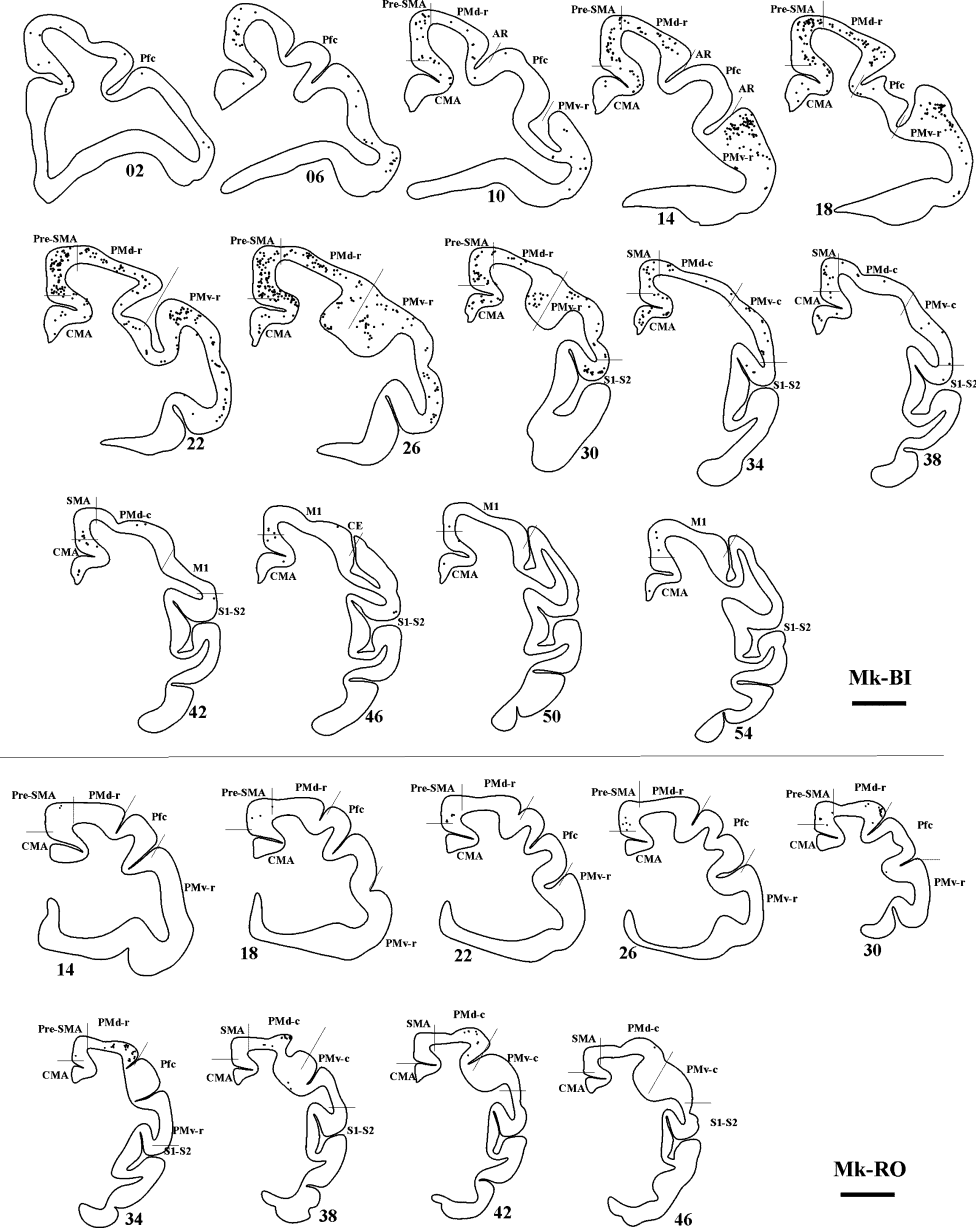
*Top panel* Frontal sections of the right hemisphere in Mk-BI (control, untreated monkey), arranged from rostral to caudal, showing the distribution of retrogradely labelled callosal neurons as a result of BDA injection in the opposite PM. The number next to each reconstruction is the serial number of the corresponding histological section. *Scale bar* 5 mm. *P* principal sulcus, *AR* arcuate sulcus, *CE* central sulcus. *Bottom panel* Frontal sections of the right hemisphere in Mk-RO (control monkey), arranged from rostral to caudal, showing the distribution of retrogradely labelled callosal neurons as a result of BDA injection in the opposite PM. Same conventions as in *top panel*

The extent and location of the BDA injections are represented by the green territories in Fig. 1a. As a result of BDA injection in PM homolateral to the M1 lesion, the distribution of retrogradely BDA-labelled callosal neurons was established in four untreated (control) monkeys, with typical results illustrated in Fig. 3 (top panel) for Mk-BI (see also Fig. 6a). PMv, pre-SMA and PMd contained the highest number of labelled neurons, although labelling was also substantial in CMA. Clearly fewer labelled neurons were present in SMA, S1–S2, prefrontal cortex (Pfc) and M1. In line with BDA injections sites covering a smaller extent of PMv as compared to PMd, the main region of origin of the callosal projections in two other control monkeys originated from the homotypic PMd in the opposite hemisphere (Fig. 3, bottom panel for Mk-RO; Mk-GE not shown). In Mk-GE, the other labelled neurons were distributed in largely comparable amounts in PMv and pre-SMA; fewer labelled neurons were found in CMA, SMA and M1. In Mk-RO (Fig. 3; bottom panel), the additional labelling outside the homotypic PMd was found in pre-SMA, whereas there was no labelling in Pfc, M1 and S1–S2. The last control monkey (Mk-CE) was subjected to a large lesion and BDA was injected in both PMd and PMv (Fig. 1a). The reconstruction of BDA retrograde labelling in the intact hemisphere (not shown) exhibited a small number of labelled callosal neurons (see Fig. 6), most of them in the homotypic region (PM) and, but to a lesser extent, in SMA.

### Callosal connectivity of PM in the anti-Nogo-A antibody-treated monkeys

**Fig. 4:**
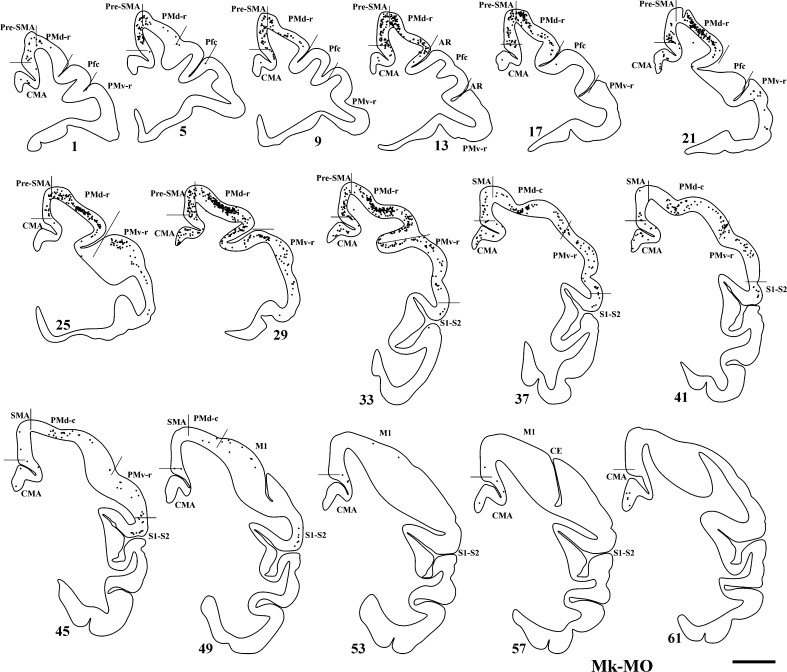
Frontal sections of the right hemisphere in Mk-MO (anti-Nogo-A antibody-treated monkey), arranged from rostral to caudal, showing the distribution of retrogradely labelled callosal neurons as a result of BDA injection in the opposite PM. *Scale bar* 5 mm. Same conventions as in Fig. 3

The distribution of BDA-labelled cells resulting from injection of BDA in the opposite PM, homolateral to the lesioned M1, assessed in three anti-Nogo-A antibody-treated monkeys (Mk-LA excluded; see Table 1), is illustrated for Mk-MO in Fig. 4. Consistent with BDA injections covering more PMd than PMv (Fig. 1a), the main zone of origin of the callosal projections was the homotypic PMd (Fig. 4), although substantial labelling was also present in pre-SMA and PMv. Additional labelled neurons were observed in SMA and CMA, besides weak labelling in Pfc, M1 and S1–S2.

**Fig. 5:**
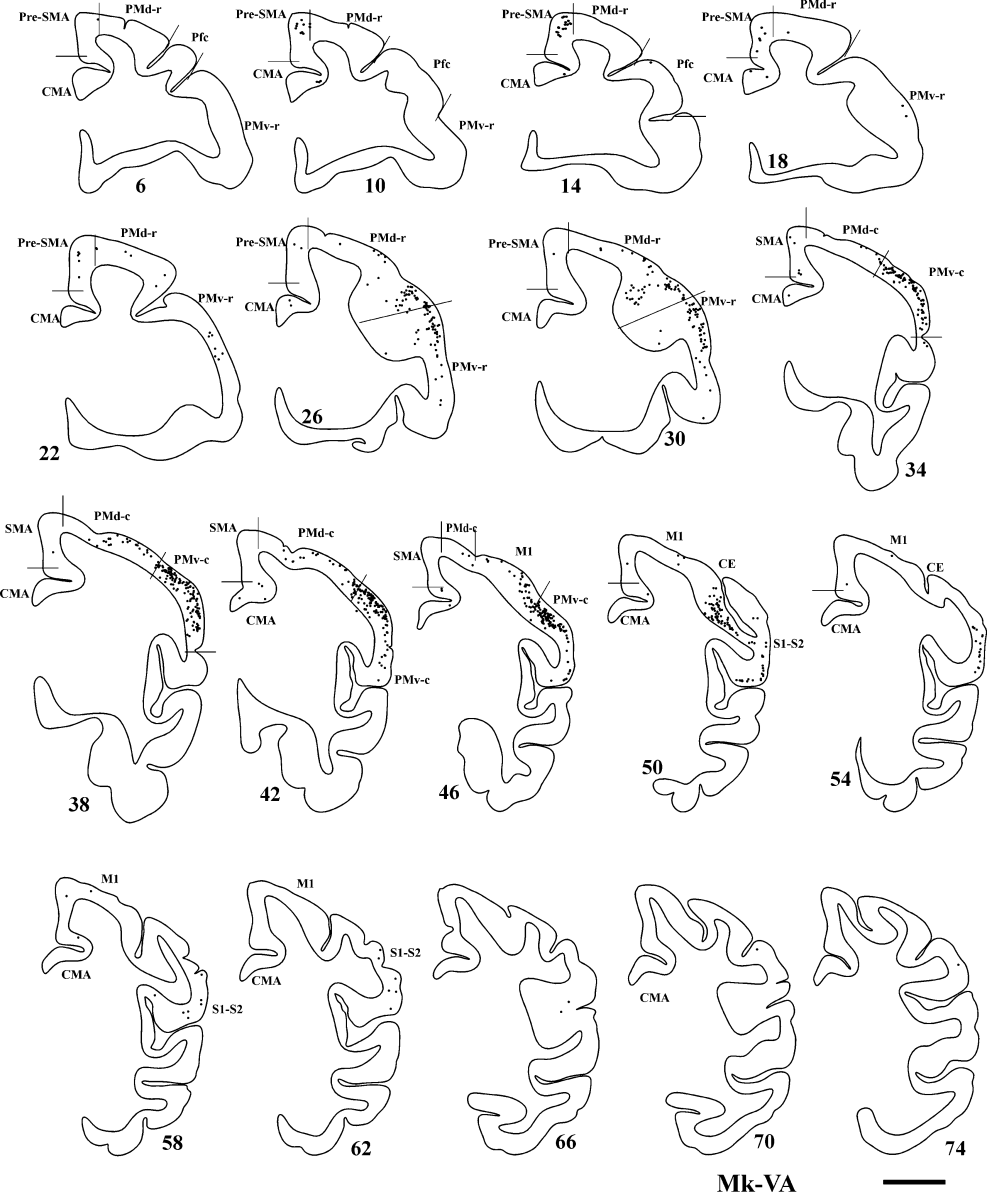
Frontal sections of the right hemisphere in Mk-VA (anti-Nogo-A antibody-treated monkey), arranged from rostral to caudal, showing the distribution of retrogradely labelled callosal neurons as a result of BDA injection in the opposite PM. Same conventions as in Fig. 3

In Mk-VA, in which the BDA injections were better balanced between PMd and PMv, the retrograde labelling was predominantly found in the homotypic PMd and PMv (Fig. 5). Other labelled neurons were located in fairly comparable amounts in pre-SMA, M1, S1–S2 and, but to a lesser extent, in SMA, Pfc and CMA.

The BDA injections performed in the ipsilesional PM in another anti-Nogo-A antibody-treated monkey (Mk-SL; not shown) yielded smaller numbers of labelled neurons. In the case of Mk-SL, most callosal projections originated from PMv, although PMd was also involved. Fewer labelled neurons were located in M1, whereas no labelling was found in CMA, Pfc, pre-SMA, SMA and S1–S2.

### Normalization of the number of labelled callosal neurons with respect to the volume of injection site

**Fig. 6:**
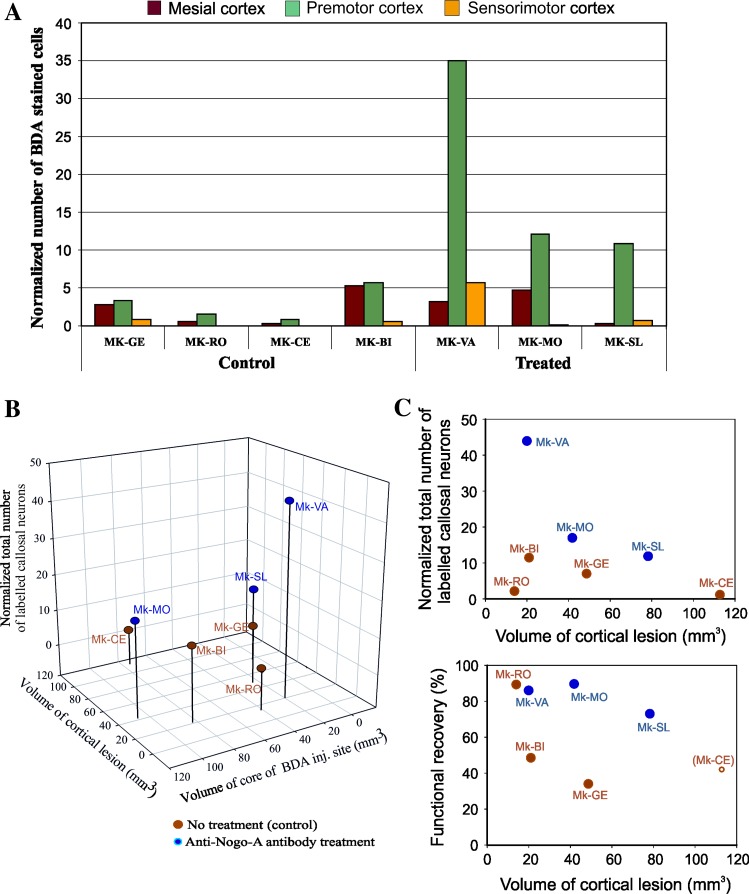
**a** Normalized number of BDA-labelled callosal neurons in the two groups of monkeys, observed in the intact hemisphere, in each case and across three main cortical zones (premotor cortex, mesial cortex and sensorimotor cortex). See text for the description of the procedure of normalization with respect to the volume of BDA injection site (core). **b** 3D plot showing the relationship of the normalized total number of labelled callosal neurons with the lesion volume and the volume of the core of the BDA injection sites (see text). **c** Two correlation plots illustrate the relationship between the normalized total number of BDA-labelled cells as a function of lesion extent (*top graph*) and between the functional recovery as a function of lesion extent (*bottom graph*). The* colour code* is the same as for **b**. In the bottom graph, the functional recovery in percent are the same values as indicated in Fig. 2, as derived from the Brinkman box task, except for Mk-CE (*empty symbol*). Mk-CE did not perform the Brinkman box task (see text), but the “modified Brinkman board” task. The latter test is generally less challenging than the Brinkman box task (Schmidlin et al. 2011). Consequently, the functional recovery value indicated for Mk-CE based on the “modified Brinkman board” task is higher than what would have been expected if Mk-CE would have performed the Brinkman box task

As there was some variability across monkeys with respect to the sites selected (based on ICMS maps post-lesion) for BDA injections, a specific analysis of the distribution of labelled cells in the distinct subregions of the various motor cortical areas would be meaningless. For this reason, only three large cortical zones were considered (Fig. 6a): the mesial cortex (pre-SMA, SMA and CMA), the premotor cortex (PM, including few neurons labelled in the prefrontal cortex) and the sensorimotor cortex (M1, post-central gyrus).

To account for the inevitable variability across monkeys due to the size of BDA injection sites (guided by ICMS maps) and the uptake of the tracer, the callosal data were normalized based on the volume of the core of the corresponding BDA injection site. The normalization was obtained by dividing the number of retrogradely labelled callosal neurons in each cortical area by the volume of the core of the injection site in mm^3^. The normalized numbers of callosal BDA-labelled neurons could thus be better compared across monkeys, in spite of the individual variability of the BDA uptake (Fig. 6a). In addition, Fig. 6a provides an estimate of the contribution of the premotor area (PMd and PMv), the mesial cortex (pre-SMA, SMA and CMA) and the sensorimotor cortex (M1, S1 and S2) to the overall callosal connectivity reaching the ipsilesional PM. The premotor area also comprises the few labelled neurons observed in the prefrontal cortex, ranging across monkeys between 0 and about 2 % of the total number of labelled cells.

In the group of control monkeys, the majority of callosal projections reaching the ipsilesional PM originated from the opposite PM, although a substantial number of projections came from the mesial cortex (Fig. 6a). The callosal projections originating from the opposite sensorimotor cortex (M1, S1 and S2) represented a fairly modest proportion. In the group of anti-Nogo-A antibody-treated monkeys (*n* = 3), there were quantitatively higher normalized numbers of callosal-labelled neurons than in the untreated monkeys (*n* = 4). There was no overlap of the normalized total number of BDA-labelled cells in the intact hemisphere between the 2 groups of monkeys (4 control versus 3 treated; see Fig. 6a, b), suggesting that the two groups are different (however, the number of monkeys in each group is too small to conduct an inter-group statistical comparison). In more detail, the anti-Nogo-A antibody-treated monkeys Mk-MO and Mk-SL yielded about twice more labelling in PM in the intact hemisphere than the untreated monkey with the highest normalized values (Mk-BI), although the BDA injection site was comparable (Mk-MO) or even smaller (Mk-SL; see Fig. 1a). In the treated monkeys Mk-VA, the amount of normalized callosal-labelled neurons reached a level close to seven times higher than in the control monkey Mk-BI. The difference was even larger when comparing the anti-Nogo-A antibody-treated monkeys Mk-VA, Mk-SL and Mk-MO with the other three control monkeys Mk-RO, Mk-CE and Mk-GE (Fig. 6a). Overall, the data presented in Fig. 6a are consistent with an increase in labelled callosal neurons in the anti-Nogo-A antibody-treated monkeys as compared to control (untreated) monkeys, all subjected to a lesion in M1. The difference is mainly due to an increase in labelling in the homotypic region (PM) of the anti-Nogo-A antibody-treated monkeys.

Although the volume of BDA injected is a major parameter influencing the present tracing data, the size of the M1 lesion may also impact on the retrograde labelling. To address this issue, the normalized total numbers of callosal neurons in the intact hemisphere were plotted for each monkey in a 3D plot, as a function of the volume of the core of the BDA injection sites and the volume of the cortical lesion affecting the grey matter (Fig. 6b). In the anti-Nogo-A antibody-treated monkeys (blue dots in Fig. 6b), the data indicate an increase in the normalized total number of labelled callosal neurons (especially in Mk-VA), as compared to the control, untreated monkeys (see also Fig. 6c). This effect is not due to larger BDA injection sites in the anti-Nogo-A antibody-treated monkeys as their sizes overlap those in the control monkeys (Fig. 6b). Similarly, the volumes of lesion in the motor cortex were in a fairly comparable (overlapping) range for the two groups of monkeys (see also Fig. 6c).

For a more comfortable representation, the normalized total number of BDA-labelled callosal neurons was plotted as a function of the volume of the cortical lesion in the form of a 2D correlative plot (Fig. 6c, top graph), indicative of an absence of correlation between these two parameters. As an extension of the preliminary behavioural data shown in Fig. 2, the percent of functional recovery post-lesion was plotted as a function of the volume of the cortical lesion (Fig. 6c, bottom graph). These data support the notion of an enhanced functional recovery in the group of anti-Nogo-A antibody-treated monkeys (blue symbols), although one control monkey (Mk-RO) recovered very well. However, Mk-RO exhibited the smallest lesion and, even in the absence of treatment, one can expect a prominent functional recovery from a restricted cortical lesion.

Besides the difference between the 2 groups of monkeys related to the normalized total number of retrogradely labelled cells in the intact hemisphere, as displayed in Fig. 6a, one may wonder whether the distribution of labelled neurons in the 3 main cortical regions (mesial cortex, premotor cortex and sensorimotor cortex) differs between the control monkeys and the anti-Nogo-A antibody-treated monkeys. To address this question, the numbers of labelled neurons in the 3 cortical regions were cumulated from the different monkeys in each group and a chi-square statistical test was performed. The result of the statistical test showed a significantly different distribution between the control and the anti-Nogo-A antibody-treated monkeys (*p* < 0.001), supporting the notion that the enhancement of callosal projection in the latter group is not homogeneous among the three cortical regions, but is rather focused on the homotypic projection from the intact PM to the ipsilesional PM.

## Discussion

### Survey of data

In the present study, we have investigated the reorganization of callosal connectivity of the ipsilesional PM following a unilateral lesion of M1 in adult macaques. The monkeys treated with the anti-Nogo-A antibody exhibited an enhanced callosal connectivity reaching the ipsilesional PM, based on the normalized numbers of retrogradely labelled neurons in the intact hemisphere, in comparison with the control (untreated) monkeys. As depicted in Fig. 6a, the enhancement of the callosal connection from the intact hemisphere to PM induced by the anti-Nogo-A antibody treatment was essentially present for the homotypic projection from PM of the intact hemisphere to the ipsilesional PM.

### Reorganization of corticocortical connectivity after lesion of M1

In a recent, very extensive and quantitative study, Dancause et al. (2005) have investigated the rearrangement of the intra-hemispheric connectivity of PM after lesion of the homolateral M1 in squirrel monkeys. It was found that projections originating from PM and directed to M1 in the intact animal were redirected to the post-central sulcus, namely to S1. Such intra-hemispheric redirection of axons was not investigated in the present study, focussed on inter-hemispheric connections. However, in line with the data of Dancause et al. (2005), one may speculate that the callosal axons originating from the intact hemisphere and directed to the lesioned M1 and perilesional territories may have been redirected in the lesioned hemisphere, maybe towards the post-central gyrus as well. This possibility could not be tested in the present material, as injections in the intact hemisphere are needed to test this hypothesis (based on anterograde labelling).

**Fig. 7:**
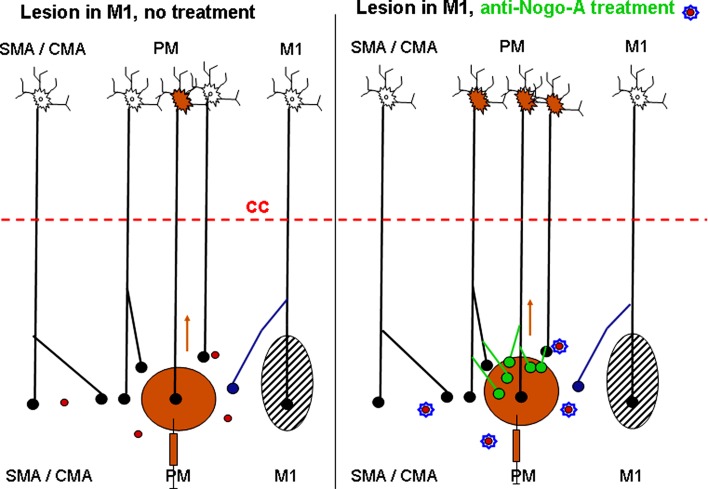
Schematic representation of the pattern of callosal projection reaching PM in monkeys subjected to a permanent lesion of M1 (*black hatched area*) and interpretation of the present results. In the *left panel*, the control (untreated) monkeys exhibit a modest, spontaneous regenerative axonal sprouting of the callosal projection reaching the ipsilesional PM (*blue* axon terminal). In the *right panel*, in contrast, the anti-Nogo-A antibody treatment promotes local compensatory sprouting of the callosal axons (*green* axon terminals), in particular in PM where BDA was injected. In other words, the results of the present study are consistent with an enhanced number of callosal neurons in the homotypic PM of the intact hemisphere in the anti-Nogo-A antibody-treated monkeys as compared to the control (untreated) monkeys. The *horizontal dashed red line* represents the corpus callosum, with the intact hemisphere above and the lesioned hemisphere below. Neurons in the intact hemisphere send callosal projections to the lesioned hemisphere, terminating with axonal boutons (*small filled circles*). The *red circles* represent Nogo-A, inhibiting axonal growth in the adult central nervous system. In the *right panel* (treated monkeys), Nogo-A is neutralized with an anti-Nogo-A antibody. The *brown syringe* represents the injection of the tracer BDA in the ipsilesional PM, whereas the *brown arrows* are for the retrograde axonal transport of BDA from the injected PM to the intact hemisphere. As a result, some neurons are retrogradely labelled with BDA (brown soma in the intact hemisphere). The area “SMA” comprises both pre-SMA and SMA-proper, whereas the area “CMA” includes the three subdivisions of CMA

The originality of the present study is essentially that the reorganization of the connectivity of PM, callosal in the present case, has been investigated in relation to a treatment (anti-Nogo-A antibody) that enhances fibre growth and plasticity in the injured and intact brain (Montani et al. 2009; Schwab 2010). As illustrated in Fig. 7 for control (untreated) monkeys (left panel), the lesion of M1 may be followed by a limited spontaneous regenerative sprouting of axons affected by the lesion (blue axon terminal). As such sprouting may be very limited in distance, injection of BDA in the ipsilesional PM may not label the corresponding neuron of origin. In contrast, in the anti-Nogo-A antibody-treated monkeys (Fig. 7, right panel), regenerative sprouting (green axon terminal arising from the blue axon terminal) may be accompanied by compensatory sprouting of intact axon terminals originating from a larger cortical zone in the intact hemisphere (other green axon terminals in right panel of Fig. 7). Some of them, especially those originating from PM, are labelled after BDA injection in the ipsilesional PM. As a result, more retrogradely labelled callosal neurons are labelled in the intact hemisphere than in control monkeys (Fig. 7). As the callosal connectivity is predominantly homotypic, the intact PM gives rise to more axon terminals in the ipsilesional PM than projections originating from other cortical areas of the intact hemisphere (Fig. 7). This interpretation is in line with the present observation (Fig. 6a) that the enhancement of callosal connectivity observed in the anti-Nogo-A antibody-treated monkeys (as compared to control monkeys) was found predominantly for the homotypic projection from the intact PM to the ipsilesional PM. Furthermore, although speculative, it may be hypothesized that reactive events in the lesion and perilesion territories stimulate sprouting, by the activation of an injury-associated molecular programme (e.g. Li et al. 2010). Such sprouting event may be limited in control monkeys, but enhanced in anti-Nogo-A antibody-treated monkeys, a phenomenon possibly more effective in territories close by the lesion (e.g. PM) than in more distant cortical areas (e.g. SMA, CMA).

As an alternative explanation to enhanced sprouting, one cannot exclude that the lesion of M1, in particular its consequences during the following hours and days (inflammation, scar formation, etc.), interrupts callosal axons reaching the ipsilesional PM, especially if the lesion encroached the white matter. A possible effect of anti-Nogo-A antibody treatment could have been to preserve some of the callosal axons from such damage, resulting in an increase in callosal connectivity in the treated monkeys. Such mechanism of preservation might have taken place in Mk-SL, in which the lesion affected significantly the white matter (subcortical territory represented in grey in Fig. 1). As indicated in Table 1, the lesion encroached the white matter in only one other monkey (Mk-CE), but to a lesser extent than Mk-SL. Overall, there is no systematic relation between the effect of anti-Nogo-A antibody treatment on callosal connectivity and the absence/presence of impact of the lesion on the white matter (Table 1). However, pro-survival effects of anti-Nogo-A antibodies or other Nogo-A suppressive treatments have never been observed up to now in a variety of different CNS lesion models, in contrast to the well-documented effects on nerve fibre sprouting and regeneration in spinal cord injury models demonstrating substantial sprouting (regenerative and compensatory), promoted by anti-Nogo-A antibody treatment (e.g. Gonzenbach and Schwab 2008; Fouad et al. 2004; Freund et al. 2007). Although not excluded, this explanation of preservation appears less likely therefore at present.

At that step, a relationship between the enhanced callosal connectivity of PM promoted by anti-Nogo-A antibody (Fig. 6b) and the improved functional recovery (Fig. 2) is not demonstrated yet. To demonstrate such an effect, it would be necessary to transiently inactivate PM in the intact hemisphere of the anti-Nogo-A antibody-treated monkeys several months after the lesion and hopefully obtain a loss of the enhanced functional recovery of the manual performance. Such reversible inactivation experiment of the intact PM was not performed in the present study, to not interfere with the retrograde labelling that was analysed in the intact PM. Reversible inactivation of PM in the intact hemisphere after unilateral lesion of M1 was, however, performed with muscimol infusion in two pilot monkeys not subjected to any treatment in our original study introducing this model of motor cortex lesion (Liu and Rouiller 1999): in line with the hypothesis of a modest sprouting of the callosal projections in control monkeys (Fig. 7, left panel), the reversible inactivation of the contralesional PM did not affect the incomplete spontaneous recovery of manual performance in the two untreated monkeys. Reversible inactivation of the intact PM is an experiment to be conducted in the future on anti-Nogo-A antibody-treated monkeys, in which there will be no analysis of BDA retrograde labelling in the intact PM.

In spite of the lesion of M1, the general pattern of callosal connectivity of PM found in the present study in both control and anti-Nogo-A antibody-treated monkeys remains generally consistent with previous reports in intact monkeys (Marconi et al. 2003; Boussaoud et al. 2005 for macaque monkeys; see also Fang et al. 2008 in a prosimian primate), with a predominance of an origin in the homotypic motor cortical area PM, a feature even accentuated as a result of the anti-Nogo-A antibody treatment. In addition, in line with these previous studies in intact monkeys, callosal inputs to the ipsilesional PM also originate from other source areas, such as pre-SMA, CMA, Pfc, SMA and, to a lesser extent, M1 and S1–S2 (Fig. 6a).

### Limitations in the interpretation of the data

First of all, as it is generally the case for non-human primate studies, the number of animals is limited, mainly for ethical reasons. Second, as each case was performed one after the other during several years, there were some adjustments in the protocol, mainly in relation to the main objective of the study dealing with the degree of functional recovery (see Table 1 for details). There is an inevitable variability across monkeys with respect to the lesion size and location (Fig. 1a; see Kaeser et al. 2010 for a more detailed discussion on that issue). While on the experimental point of view, reproducible lesion sizes would be optimal, on the other hand, the significant variability of lesion sizes in M1 observed in the present study is more representative of the even larger variability of cortical lesions observed in human subjects (see also a recent non-human primate model of motor cortex lesion, performed surgically and yielding variable lesion extent as well: Darling et al. 2009). The present experimental model of motor cortex lesion in non-human primates, including significant inter-individual variability in term of lesion size, may thus be more pertinent for evaluating the translational value of a treatment towards clinics.

Other parameters are the location and size of BDA injections, as well as the efficacy of BDA uptake, which were only in part compensated in the present study by normalizing the number of retrogradely labelled callosal neurons by the volume of the injection site (core; see Fig. 6). Furthermore, in Mk-SL for instance, the lesion spread largely subcortically in the white matter (see Fig. 1a), more than in the other monkeys, having possibly interrupted callosal fibres. As a result, the number of labelled callosal neurons in Mk-SL was especially low in spite of a large volume of BDA injected. The normalization based on dividing the number of labelled cells by the volume of the injection site (core), as proposed here, represents only a first approximation, in order to compensate tentatively for inter-individual variability of tracer uptake. A linear relationship between the number of labelled cells and the volume of the injection site (core) is unlikely, considering that injection sites of similar volume may spread differently across the grey matter versus white matter and, in the grey matter, differently across the various cortical layers exhibiting variable densities of callosal terminals. An optimal normalization should thus comprise a large palette of parameters, some of them being especially difficult to assess quantitatively. Nevertheless, the present normalization based on the volume of the injection site is largely consistent with a normalization based on the volume of BDA injected (not shown).

Another variable parameter is the duration of survival time after BDA injection (Table 1). Nevertheless, there was no systematic difference of survival times between the 2 groups (e.g. short survival times in control monkeys and long survival times in treated monkeys, or vice versa), which may explain the increase in retrograde BDA labelling in the anti-Nogo-A antibody-treated monkeys. The absence of effect of survival time duration on BDA labelling, above a minimal duration required for the transport of the tracer to cover the desired distance, is in line with previous studies using BDA (Rouiller et al. 1999; Tanné-Gariépy et al. 2002a, b; Liu et al. 2002; Morel et al. 2005; Boussaoud et al. 2005).

## Conclusion

In spite of the above limitations of interpretation, the present study supports the hypothesis that, after lesion of M1, the callosal projections reaching the ipsilesional PM are enhanced after anti-Nogo-A antibody treatment. The proposed mechanism is an enhancement of sprouting of callosal axons near their target, promoted by the neutralization of the neurite growth inhibitor Nogo-A (see Fig. 7). Such a mechanism of axonal sprouting near a lesioned territory (PM in that case) is comparable to the increased sprouting of CS axons observed in spinal cord injured monkeys treated with the anti-Nogo-A antibody, as compared to control antibody-treated monkeys (Fouad et al. 2004; Freund et al. 2006, 2007). Future analyses are needed to determine whether projections to PM, other than callosal ones, are also promoted by the anti-Nogo-A antibody treatment in monkeys with lesion of M1, such as the thalamocortical projection for instance. Moreover, specific reversible inactivation experiments will be necessary to demonstrate that the reorganization of connections promoted by a treatment is indeed the support of enhanced functional recovery, a demonstration still lacking at that step for the PM callosal projections reported in the present study.

As far as the mechanisms of functional recovery from M1 lesion are concerned, in addition to the possible role (still to be demonstrated) of connectional reorganization at cortical level suggested here and in a previous study (Dancause et al. 2005), plasticity at subcortical level is likely to play a role as well, for instance the projection of motor cortical areas preserved by the lesion to the brainstem (to mobilize the reticulospinal system for instance) and, of course, the corticospinal projection system. It was indeed shown that after lesion of the motor cortex and lateral premotor cortex, the corticospinal projection originating from the intact ipsilesional SMA exhibited sprouting in the cervical cord (McNeal et al. 2010). In the present study, the infusion of anti-Nogo-A antibody at cervical level (in addition to delivery at cortical level near the lesion) may have enhanced such intraspinal axonal sprouting, a mechanism to test in future functional investigations.

## Footnotes

1 A new Swiss regulation has been introduced in September 2010 requesting now a volume of 45 m^3^ at least to be given to a group of up to 5 macaque monkeys.
